# Neutrophil-percentage-to-albumin ratio is associated with chronic kidney disease: Evidence from NHANES 2009–2018

**DOI:** 10.1371/journal.pone.0307466

**Published:** 2024-08-05

**Authors:** Jinxi Li, Ting Xiang, Xinyun Chen, Ping Fu

**Affiliations:** 1 Department of Nephrology, Kidney Research Institute, West China Hospital, Sichuan University, Chengdu, Sichuan Province, China; 2 Department of Health Management, Health Management Center, General Practice Center, West China Hospital, Sichuan University, Chengdu, Sichuan Province, China; University of Utah School of Medicine, UNITED STATES OF AMERICA

## Abstract

**Introduction:**

The neutrophil-percentage-to-albumin ratio (NPAR), a novel inflammatory biomarker, has been used to predict the prognosis of patients with cancer and cardiovascular disease. However, the relationship between NPAR and chronic kidney disease (CKD) remains unknown. The purpose of this study was to investigate the possible association between NPAR and CKD.

**Methods:**

The cross-sectional study included participants with complete information on NPAR, serum creatinine (Scr), or urinary albumin-to-creatinine ratio (UACR) from the 2009–2018 National Health and Nutrition Examination Survey (NHANES). CKD was defined as the presence of either low estimated glomerular filtration rate (eGFR) or albuminuria. Univariate and multivariate logistic regression and restricted cubic spline regression were used to assess the linear and nonlinear associations between NPAR and renal function. Subgroup and interactive analyses were performed to explore potential interactive effects of covariates. Missing values were imputed using random forest.

**Results:**

A total of 25,236 participants were enrolled in the study, of whom 4518 (17.9%) were diagnosed with CKD. After adjustment for covariates, the odds ratios (ORs) for prevalent CKD were 1.19 (95% CI = 1.07–1.31, *p* <0.05) for the Q2 group, 1.53 (95% CI = 1.39–1.69, *p* < 0.001) for the Q3 group, and 2.78 (95% CI = 2.53–3.05, *p* < 0.001) for the Q4 group. There was a significant interaction between age and diabetes mellitus on the association between NPAR and CKD (both *p* for interaction < 0.05). And there was a non-linear association between NPAR levels and CKD in the whole population (*p* for non-linear < 0.001). All sensitivity analyses supported the positive association between NPAR and CKD.

**Conclusions:**

NPAR was positively correlated with increased risk of CKD. The NPAR may serve as an available and cost-effective tool for identifying and intervening the individuals at risk of CKD.

## 1 Introduction

Chronic kidney disease (CKD) is defined as abnormalities in kidney structure or function that persist for three months or more and have a significant impact on health [1], and is characterized by a decrease in estimated glomerular filtration rate (eGFR) or an increase in urinary albumin levels [2]. CKD is a major contributor to global mortality and represents a significant burden to society [3]. Study shown that more than 843.6 million patients have CKD in 2017, and this number is expected to increase [4]. Additionally, CKD would be the fifth leading cause of death by 2040 [5]. Inflammation plays a critical role in the development and progression of CKD and is strongly associated with increased mortality [6]. Research has shown that inflammatory factors reduce renal blood flow and promote renal fibrosis [7]. Biomarkers of inflammation have potential predictive value in CKD. Cohort studies have suggested that biomarkers of inflammation are negatively related with renal function, but positively associated with albuminuria [8]. Therefore, the search for specific biomarkers for the early diagnosis of CKD is imminent, which is beneficial to further prevention.

The neutrophil-percentage-to-albumin ratio (NPAR) is a novel index that reflects systemic inflammatory and immunologic conditions [9]. The NPAR takes into account neutrophil percentage and albumin in the blood. Accumulating evidence has shown that NPAR was widely used to assess the risk and prognosis of diseases. For example, Cui et al. found that the high NPAR was associated with in-hospital mortality in patients with ST-segment elevation myocardial infarction [10]. In addition, there were close association between NPAR level and the increased risk of all-cause mortality in patients with acute kidney injury (AKI) [11]. However, due to the limited number of studies, the relationship between CKD and NPAR remains scarce and requires further investigation. In this study, we aimed to investigate the relationship between NPAR and CKD in participants of the National Health and Nutrition Examination Survey (NHANES).

## 2 Materials and methods

### 2.1 Study population

In this study, we extracted data from the NHANES, which is based on a nationally representative cross-sectional design. The purpose of the NHANES is to assess the nutritional and health status of the United States (US) population through interviews, physical examinations, and laboratory tests, and the data are updated every 2 years. The survey was approved by the National Center for Health Statistics Research Ethics Review Board, and participants signed informed consent forms. The detailed NHANES study design and data are available at https://www.cdc.gov/nchs/nhanes/.

**Fig 1:**
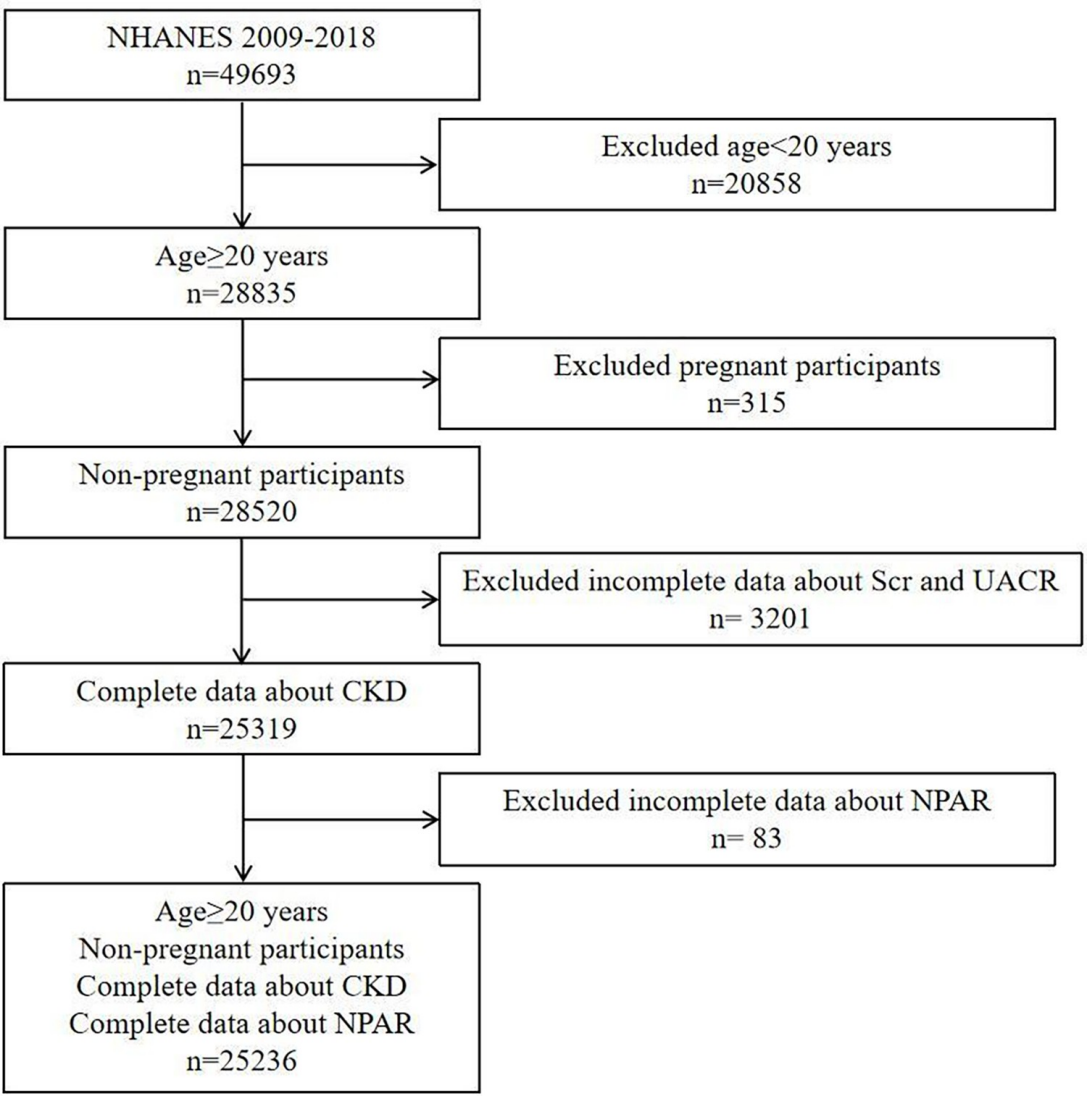
A flow chart of the sample selection from NHANES 2009–2018. Scr, serum creatinine; UACR, urinary albumin to creatinine ratio; CKD, chronic kidney disease. NPAR, neutrophil-percentage-to-albumin ratio.

This study selected five NHANES cycles from 2009 to 2018 to assess the association between NPAR and CKD. A total of 49,693 participants were initially enrolled. After excluding participants aged <20 years (n = 20,858), participants who were pregnant (n = 315), participants with incomplete data on serum creatinine (Scr) or urinary albumin-to-creatinine ratio (UACR) (n = 3201), and participants with incomplete data on NPAR (n = 83), 25,236 participants were included in our final analysis (**Fig 1**).

### 2.2 Exposure variables

White blood cell counts were measured by using an automated hematology analyzer (Coulter® DxH 800 analyzer). The Coulter VCS system was used for measuring the neutrophil percentage. NPAR was used as the exposure variable in our analysis. NPAR was calculated by the following formula: neutrophil percentage (%) / albumin (g/dL).

### 2.3 Outcome variable

The diagnostic criteria for CKD were based on the 2012 Kidney Disease: Improving Global Outcomes (KIDGO) recommendations—the presence of either low estimated glomerular filtration rate (eGFR) or albuminuria [12]. The Chronic Kidney Disease Epidemiology Collaboration study (CKD-EPI) equation was used to calculate eGFR according to serum creatinine (Scr) level [13]. An eGFR of less than 60 mL/min/1.73 m^2^ was defined as low. A UACR of 30 mg/g or higher was considered as albuminuria. Urinary albumin was measured by solid phase fluorescence immunoassay, and urinary creatinine was measured by an enzymatic method [14].

### 2.4 Covariates

Potential covariates that might confound the association between NPAR and CKD were selected in the multivariable-adjusted models. Covariates included: age, gender, race, marital status, education level, poverty income ratio (PIR), alcohol consumption, smoking status, BMI, physical activity, cancer, gout, cardiovascular disease (CVD), high blood pressure (HBP), diabetes mellitus [8], albumin, blood urea nitrogen (BUN), serum uric acid (UA), serum glucose, Hbalc, triglyceride [2], low-density lipoprotein (LDL), high-density lipoprotein (HDL), total cholesterol (TC), UACR, eGFR, hemoglobin (HGB), neutrophil count (NC), neutrophil percentage, CKD.

Race was categorized into five groups: Mexican American, other Hispanic, non-Hispanic white, non-Hispanic black and other. Marital status was divided into six categories: married, widowed, divorced, separated, never married, and living with a partner. Education level was divided into three categories: less than high school, high school, and more than high school. PIR was divided into three categories: low (<1.5), medium (1.5–3.5) and high (>3.5). Alcohol consumption was dichotomized as never (had <12 drinks in lifetime), low-to-moderate (≤1 drink per day for women or ≤2 drinks per day for men average over the past year) and severe (>1 drink per day for women or >2 drinks per day for men averaged over the past year). Smoking status was categorized into three groups: never smokers (smoked <100 cigarettes in lifetime), former smokers (smoked >100 cigarettes in lifetime and do not smoke now), and current smokers (smoked >100 cigarettes in lifetime and smoke some days or every day). Physical activity was divided into four categories according to work activity. Participants who ever reported having congestive heart failure, coronary heart disease, angina/angina pectoris and heart attack were diagnosed as having CVD. Participants were defined as having HBP if they had systolic blood pressure ≥130 mmHg or diastolic blood pressure ≥90 mmHg, a physician diagnosis of HBP, or use of antihypertensive medication. To be consistent with the diagnostic criteria for DM, we included an expanded diagnostic cluster using six reference definitions: physician diagnosis of DM, use of antidiabetic medication or insulin, fasting glucose ≥7.0 mmol/L, 2-hour blood glucose ≥11.1 mmol/L during oral glucose tolerance test, and glycohemoglobin (HbA1c) ≥6.5%.

### 2.5 Statistical analyses

All statistical analyses were performed according to the NHANES recommendations. Continuous variables were presented as mean with SD or median with interquartile range, and categorical variables were presented as frequencies with percentages. Student’s t-test (continuous variables) or chi-squared test (categorical variables) was used to calculate differences in baseline characteristics between different NPAR groups (quartiles). Restricted cubic spline (RCS) was used to determine the optimal cutoff value of the NPAR level. The association between NPAR and CKD in different models was evaluated by multivariate logistic regression analysis. Model 1: No adjustment for confounders. Model 2: Age, gender and race were adjusted. Model 3: age, gender, race, marital status, education level, PIR, alcohol consumption, smoking status, BMI, HBP, DM, and CVD were adjusted. In addition, given that there were 0–15% missing values in various covariates, we interpolated the dataset using multivariate imputation by chained equations based on random forest methods. Subgroup analysis was performed to examine the relationship between NPAR and CKD in different subgroups. Stratification factors included age (<60/≥60 years), gender (male/female), race (black/other), HBP (yes/no), diabetes (yes/no), CVD (yes/no), BMI (<25/≥25 kg/m^2^). Interaction analysis was added to assess heterogeneity of association between subgroups. All statistical analyses were performed using R, version 4.2.0 (http://www.R-project.org, The R Foundation). Results were considered statistically significant at a two-sided *p* < 0.05.

## 3 Results

### 3.1 Baseline characteristics of included participants

A total of 25,236 participants were included in this study with a mean age of 49.69 years, of which 48.9% were male. 4518 (17.9%) participants were diagnosed with CKD according to eGFR and UACR. The mean eGFR was 93.85 mL/min/1.73 m^2^. The median and IQR of the UACR was 7.19 (9.31) mg/g. The demographic and clinical characteristics of the participants by NPAR quartiles are shown in **Table 1**, which shows statistically significant differences in race, marital status, education level, PIR, alcohol consumption, smoking status, BMI, physical activity, cancer, gout, CVD, HBP, DM, albumin, BUN, UA, serum glucose, Hbalc, TG, LDL, HDL, TC, HGB, NC and neutrophil percentage.

**Table 1:** Baseline characteristics of included participants (n = 25236) in the NHANES 2009–2018.

Variable	Overall	Neutrophil-percentage-to-albumin ratio in Quartile				P-value
		Q1	Q2	Q3	Q4	
	n = 25236	n = 6316	n = 6306	n = 6313	n = 6301	
Male, n (%)	12360 (48.9)	3545 (28.7)	3188 (25.8)	2950 (23.9)	2677 (21.7)	<0.001
Age (years)	49.69 ± 17.54	46.54 ± 17.20	47.75 ± 16.97	50.49 ± 17.25	54.02 ± 17.79	<0.001
Race, n (%)						<0.001
Mexican American	3756 (14.9)	752 (20.0)	1029 (27.4)	1025 (27.3)	950 (25.3)	
Other Hispanic	2653 (10.5)	635 (23.9)	693 (26.1)	671 (25.3)	654 (24.7)	
Non-Hispanic white	10069 (39.9)	1975 (19.6)	2446 (24.3)	2721 (27.0)	2927 (29.1)	
Non-Hispanic black	5224 (20.7)	1924 (36.8)	1159 (22.2)	1047 (20.0)	1094 (20.9)	
Others	3534 (14.0)	979 (27.7)	879 (24.0)	849 (24.0)	676 (19.1)	
Marital status, n (%)						<0.001
Married	12909 (51.2)	3156 (24.4)	3288 (25.5)	3341 (25.9)	3124 (24.2)	
Widowed	1930 (7.6)	366 (19.0)	396 (20.5)	464 (24.0)	704 (36.5)	
Divorced	2778 (11.0)	611 (22.0)	690 (24.8)	715 (25.7)	762 (27.4)	
Separated	865 (3.4)	180 (20.8)	216 (25.0)	228 (26.4)	241 (27.9)	
Never married	4674 (18.5)	1439 (30.8)	1169 (25.0)	1038 (22.2)	1028 (22.0)	
Living with partner	2080 (8.2)	564 (27.1)	547 (26.3)	527 (25.3)	442 (21.2)	
Educational level, n (%)						<0.001
Less than high school	5897 (23.4)	1373 (23.3)	1436 (24.4)	1494 (25.3)	1594 (27.0)	
High school	5645 (22.4)	1378 (24.4)	1392 (24.7)	1435 (25.4)	1440 (25.5)	
More than high school	13694 (54.2)	3565 (26.0)	3478 (25.4)	3384 (24.7)	3267 (23.9)	
PIR, n (%)						<0.001
Low	9342 (37.0)	2277 (24.4)	2212 (23.7)	2381 (25.5)	2472 (26.5)	
Moderate	8213 (32.5)	2013 (24.5)	2046 (24.9)	2051 (25.0)	2103 (25.6)	
High	7681 (30.4)	2026 (26.4)	2048 (26.7)	1881 (24.5)	1726 (22.5)	
Alcohol consumption, n (%)						0.012
No	7699 (30.5)	1901 (24.7)	1856 (24.1)	1896 (24.6)	2046 (26.6)	
Low-to-moderate	15535 (61.6)	3902 (25.1)	3944 (25.4)	3919 (25.2)	3770 (24.3)	
Severe	2002 (7.9)	513 (25.6)	506 (25.3)	498 (24.9)	485 (24.2)	
Smoking status, n (%)						<0.001
Never	14257 (56.5)	3765 (26.4)	3745 (26.3)	3550 (24.9)	3197 (22.4)	
Former	5974 (23.7)	1378 (23.1)	1384 (23.2)	1510 (25.3)	1702 (28.5)	
Current	5005 (19.8)	1173 (23.4)	1177 (23.5)	1253(25.0)	1402 (28.0)	
BMI, kg/m^2^	29.30 ± 7.01	27.86 ± 5.91	28.58 ± 6.35	29.52 ± 6.82	31.24 ± 8.27	<0.001
Physical activity, n (%)						<0.001
Inactive	14997 (59.4)	3730 (24.9)	3658 (24.4)	3745 (25.0)	3864 (25.8)	
Moderate	5256 (20.8)	1255 (23.9)	1365 (26.0)	1319 (25.1)	1317 (25.1)	
Vigorous	1084 (4.3)	293 (27.0)	265 (24.4)	279 (25.7)	247 (22.8)	
Moderate and vigorous	3899 (15.5)	1038 (26.6)	1018 (26.1)	970 (24.9)	873 (22.4)	
Cancer, n (%)	2350 (9.3)	416 (17.7)	490 (20.9)	610 (26.0)	834 (35.5)	<0.001
Gout, n (%)	1169 (4.6)	226 (19.3)	253 (21.6)	286 (24.5)	404 (34.6)	<0.001
CVD, n (%)	2099 (8.3)	345 (16.4)	422 (20.1)	523 (24.9)	809 (38.5)	<0.001
HBP, n (%)	9194 (36.4)	1964 (21.4)	2071 (22.5)	2318 (25.2)	2841 (30.9)	<0.001
DM, n (%)	4876 (19.3)	899 (18.4)	1011 (20.7)	1244 (25.5)	1722 (35.3)	0.011
Albumin, g/dL	42.33 ± 3.40	42.50 ± 3.35	42.60 ± 3.24	42.42 ± 3.36	41.80 ± 3.59	<0.001
BUN, mmol/L	4.98 ± 2.14	4.76 ± 1.75	4.86 ± 1.78	4.95 ± 2.06	5.33 ± 2.75	<0.001
Scr, μmol/L	75.14 (62.76–88.40)	76.91 (65.42-,89.28)	74.26 (62.76–88.40)	73.37 (61.88–87.52)	74.26 (61.88–91.05)	<0.001
UA, μmol/L	324.74 ± 86.13	326.77 ± 84.07	323.66 ± 84.10	321.48 ± 84.01	327.05 ± 91.97	<0.001
Glucose, mmol/L	5.61 (5.22–6.22)	5.55 (5.16–6.00)	5.51 (5.22–6.11)	5.61 (5.22–6.22)	5.76 (5.27–6.49)	<0.001
Hbalc, mg/dl	5.79 ± 1.12	5.68±0.95	5.69±0.95	5.81±1.14	6.00±1.34	<0.001
TG, mmol/L	1.13 (0.79–1.69)	1.08 (0.73–1.67)	1.13 (0.79–1.71)	1.15 (0.81–1.69)	1.16 (0.82–1.68)	<0.001
LDL, mmol/L	2.94 ± 0.92	3.01 ± 0.95	2.99 ± 0.93	2.94 ± 0.91	2.81 ± 0.90	<0.001
HDL, mmol/L	1.29 (1.09–1.60)	1.32 (1.09–1.63)	1.29 (1.09–1.60)	1.29 (1.09–1.58)	1.27 (1.06–1.55)	<0.001
TC, mmol/L	4.95 ± 1.07	5.04 ± 1.09	5.01 ± 1.08	4.94 ± 1.03	4.80 ± 1.05	<0.001
UACR, mg/g	7.19 (4.63–13.94)	6.31 (4.16–10.91)	6.79 (4.50–12.13)	7.40 (4.73–14.18)	8.95 (5.32–21.60)	<0.001
eGFR, mL/min/1.73m^2^	93.85 ± 23.58	97.41 ± 21.71	95.82 ± 22.29	93.70 ± 22.79	88.45 ± 26.29	<0.001
HGB, g/L	14.00 ± 1.52	14.17 ± 1.45	14.13 ± 1.47	14.02 ± 1.48	13.68 ± 1.62	<0.001
NC, ×10^9^ cell/L	4.0 (3.1–5.1)	2.8 (2.2–3.5)	3.7 (3.1–4.4)	4.3 (3.6–5.2)	5.4 (4.4–6.6)	<0.001
Neutrophil percentage, %	57.70 ± 9.49	46.33 ± 6.57	55.62 ± 4.18	61.00 ± 4.31	67.85 ± 5.81	<0.001
CKD, n (%)	4518 (17.9)	769 (17.0)	890 (19.7)	1106 (24.5)	1753 (38.8)	<0.001

PIR, poverty income ratio; BMI, body mass index; CVD, cardiovascular disease; HBP, high blood pressure; DM, diabetes mellitus; BUN, blood urea nitrogen; Scr, serum creatinine; UA, uric acid; TG, triglyceride; LDL, low-density lipoprotein; HDL, high-density lipoprotein, TC, total cholesterol; UACR, urinary albumin creatinine ratio; eGFR, estimated glomerular filtration rate; HGB, hemoglobin; NC, neutrophil count; CKD, chronic kidney disease.

### 3.2 The cross-sectional association between the NPAR and kidney function

In univariate analysis, participants with a higher level of NPAR had a higher risk of having CKD (OR = 1.178, 95% CI: 1.164–1.193, *p* < 0.001, **Table 2**). After adjusting for other potential confounders, we created a series of models to assess the independent effects of NPAR on CKD, and high NPAR levels were still an independent risk factor for CKD (OR = 1.126, 95% CI: 1.111–1.141, *p* < 0.001). NPAR quartiles were then used. In the crude model, the OR for the Q2 group was 1.19 (95% CI = 1.07–1.31, *p* < 0.01), the OR for the Q3 group was 1.53 (95% CI = 1.39–1.69, *p* < 0.001), and that for the Q4 group was 2.78 (95% CI = 2.53–3.05, *p* < 0.001). In the full model (model 3), the OR for the Q2 group was 1.14 (95% CI = 1.01–1.27, *p* < 0.05), the OR for the Q3 group was 1.25 (95% CI = 1.22–1.40, *p* < 0.001), and that for the Q4 group was 1.86 (95% CI = 1.68–2.07, *p* < 0.001). All three models supported the increased risk of CKD with a high NPAR (all *p* for trend < 0.001).

**Table 2:** The association between NPAR and CKD.

Models	NPAR (Continuous)	NPAR (As Quartiles)				
	OR (95% CI)	Q1 (Reference)	Q2 Group OR (95% CI)	Q3 Group OR (95% CI)	Q4 Group OR (95% CI)	*p* for Trend
Model1	1.178 (1.164–1.193) ***	1.00	1.19 (1.07–1.31) **	1.53 (1.39–1.69) ***	2.78 (2.53–3.05) ***	<0.001
Model2	1.126 (1.111–1.141) ***	1.00	1.18 (1.05–1.31) **	1.35 (1.21–1.50) ***	2.15 (1.94–2.38) ***	<0.001
Model3	1.099 (1.084–1.114) ***	1.00	1.14 (1.01–1.27) *	1.25 (1.12–1.40) ***	1.86 (1.68–2.07) ***	<0.001

OR: odds ratio. 95% CI: 95% confidence interval.

* *p* < 0.05

** *p* < 0.01

*** *p* < 0.001

Model 1: crude model. Model 2: adjusted for demographic characteristics including age, gender, and race. Model 3: further adjusted for age, gender, race, marital status, educational level, PIR, alcohol consumption, smoking status, BMI, HBP, DM, and CVD.

According to the definition of CKD, individuals were classified based on their eGFR and ACR levels respectively. The low eGFR group comprised individuals with an eGFR less than 60 mL/min/1.73m^2^. In the adjusted model (model 3), increasing NPAR levels were found to be associated with an increased risk of low eGFR (OR = 1.085, 95% CI: 1.065–1.106, *p* < 0.001, as shown in **Table 3**). As a categorical variable, the OR for the Q4 group was 1.66 (95% CI = 1.42–1.93, *p* < 0.001).

**Table 3:** The association between NPAR and low eGFR.

Models	NPAR (Continuous)	NPAR (As Quartiles)				
	OR (95% CI)	Q1 (Reference)	Q2 Group OR (95% CI)	Q3 Group OR (95% CI)	Q4 Group OR (95% CI)	*p* for Trend
Model1	1.205 (1.185–1.225) ***	1.00	1.28 (1.11–1.49) **	1.59 (1.38–1.84) ***	3.18 (2.79–3.63) ***	<0.001
Model2	1.110 (1.089–1.0130) ***	1.00	1.24 (1.05–1.47) **	1.20 (1.03–1.41) *	1.90 (1.64–2.21) ***	<0.001
Model3	1.085 (1.065–1.106) ***	1.00	1.21 (1.02–1.43) *	1.12 (0.95–1.31)	1.66 (1.42–1.93) ***	<0.001

OR: odds ratio. 95% CI: 95% confidence interval.

* *p* < 0.05

** *p* < 0.01

*** *p* < 0.001.

Model 1: crude model. Model 2: adjusted for demographic characteristics including age, gender, and race. Model 3: further adjusted for age, gender, race, marital status, educational level, PIR, alcohol consumption, smoking status, BMI, HBP, DM, and CVD.

Individuals with UACR greater than 30 mg/g were categorized into the proteinuria group. The increase in NPAR levels was accompanied by a higher risk of proteinuria after adjusting with other confounding factors (OR = 1.112, 95% CI: 1.095–1.129, *p* < 0.001, as shown in **Table 4**). Besides, in model 3, the OR for the Q4 group was 2.05 (95% CI = 1.82–2.31, *p* < 0.001).

**Table 4:** The association between NPAR and proteinuria.

Models	NPAR (Continuous)	NPAR (As Quartiles)				
	OR (95% CI)	Q1 (Reference)	Q2 Group OR (95% CI)	Q3 Group OR (95% CI)	Q4 Group OR (95% CI)	*p* for Trend
Model1	1.173 (1.156–1.190) ***	1.00	1.15 (1.02–1.30) *	1.54 (1.37–1.73) ***	2.72 (2.44–3.03) ***	<0.001
Model2	1.141 (1.125–1.158) ***	1.00	1.16 (1.03–1.32) *	1.47 (1.30–1.65) ***	2.36 (2.11–2.65) ***	<0.001
Model3	1.112 (1.095–1.129) ***	1.00	1.12 (0.99–1.28)	1.36 (1.21–1.54) ***	2.05 (1.82–2.31) ***	<0.001

OR: odds ratio. 95% CI: 95% confidence interval.

* *p* < 0.05

** *p* < 0.01

*** *p* < 0.001.

Model 1: crude model. Model 2: adjusted for demographic characteristics including age, gender, and race. Model 3: further adjusted for age, gender, race, marital status, education level, PIR, alcohol consumption, smoking status, BMI, HBP, DM, and CVD.

### 3.3 Association between the NPAR and CKD subgroup and interactive analyses

**Fig 2:**
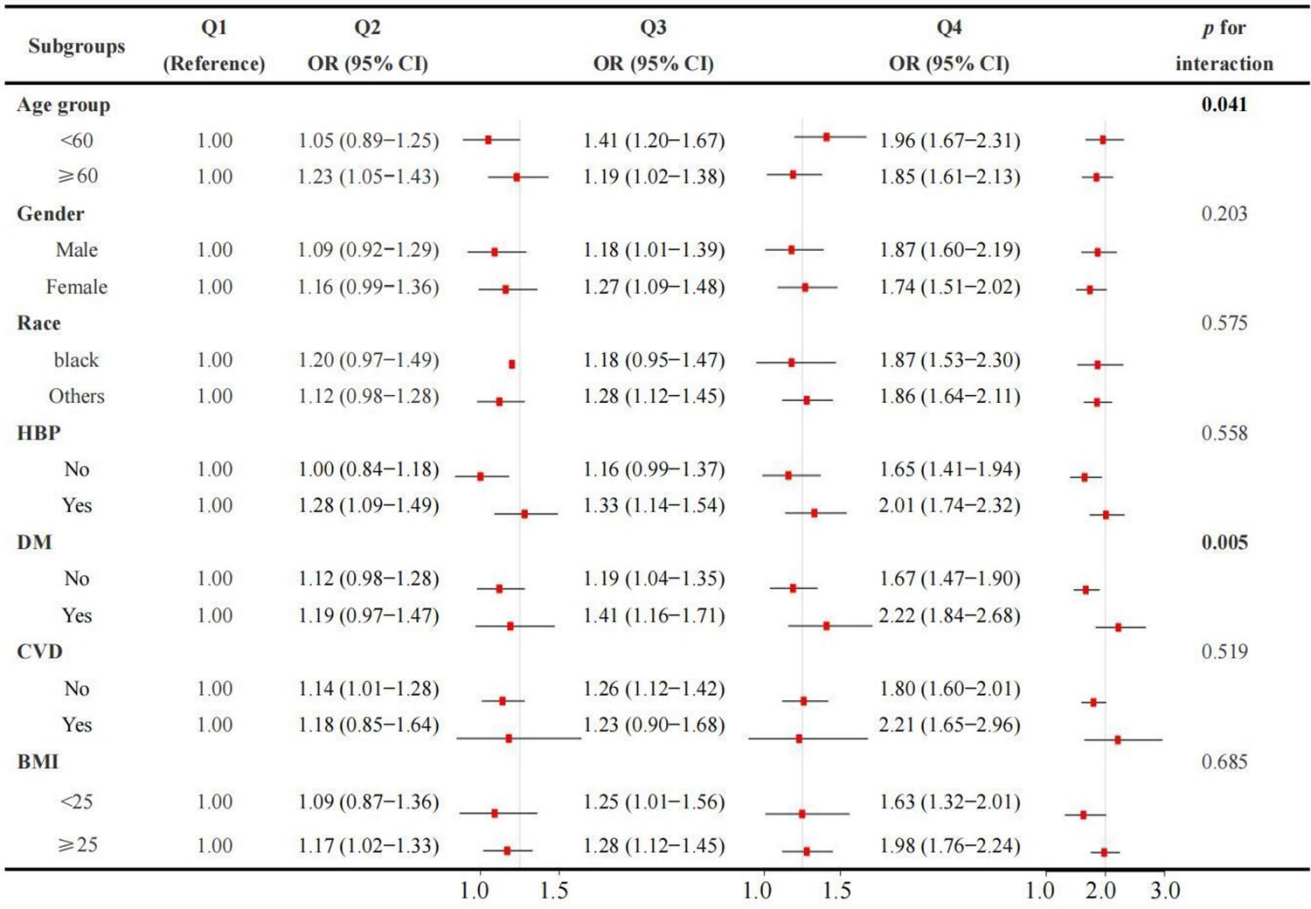
Association between NPAR and CKD in subgroup and interactive analyses. The Q1 group was used as the reference group. In the multivariable logistic regression models, covariates were adjusted as model 3 in previous analyses except for subgroup variables.

The result of subgroup analysis results suggested a consistent relationship between NPAR levels and CKD. **Fig 2** showed that most subgroups had a significant increase in the risk of CKD. Interaction tests revealed that the relationship between NPAR and CKD was not statistically different across strata, indicating that gender, race, HBP, CVD, and BMI did not significantly impact this positive correlation (*p* for interaction > 0.05). Notably, there was a significant interaction between age and DM on the association between NPAR and CKD (both *p* for interaction < 0.05).

### 3.4 The dose-response association between NPAR and CKD

**Fig 3:**
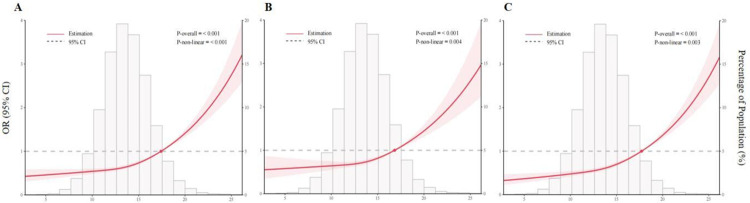
The dose–response association between the NPAR and kidney function. The dose-response relationship was evaluated using restricted cubic spline regression for the NPAR and CKD (A), low eGFR (B), and proteinuria (C), with covariates adjusted as in model 3. The odds ratio is represented by the red line and the 95% confidence interval is shown in pink.

An RCS regression was conducted to investigate the dose-response association between NPAR and CKD. **Fig 3A** showed a non-linear association between NPAR levels and CKD in the overall population (*p* for non-linear < 0.001). Additionally, we found a non-linear association between continuous NPAR levels and low eGFR (*p* for non-linear = 0.004, **Fig 3B**), as well as a non-linear association between NPAR levels and proteinuria (*p* for non-linear = 0.003, **Fig 3C**).

## 4 Discussion

Compared to the traditional clinical risk factors associated with CKD, the neutrophil-percentage-to-albumin ratio (NPAR) has received less attention in public health. This cross-sectional study enrolled 25,236 participants and firstly found a positive correlation between the increasing NPAR levels and the higher CKD morbidity, which may provide a novel biomarker for clinical diagnosis in the future.

Our study found that elevated NPAR levels were associated with a higher risk of CKD, low eGFR and proteinuria. Even when NPAR was divided into quartiles, a significant trend was also observed with increasing NPAR levels and decreasing renal function. Subgroup and interaction analyses showed that these associations were consistent across different population settings, except for age and DM. In addition, a non-linear relationship between NPAR and CKD was observed in the RCS analysis.

Consistent with our findings, the higher NPAR levels are associated with the higher incidence of diseases, such as heart failure [15], coronary artery disease [16], and ST-segment elevation myocardial infarction [10]. NPAR levels were also closely related to the complications of diabetes. DM patients with higher NPAR level had higher risk of diabetic retinopathy [9]. In addition, high NPAR levels increased the risk of 30-, 90-, and 365-day all-cause mortality in critically ill patients with AKI [11]. In fact, DM and AKI are closely related to the development of CKD. Therefore, we propose to speculate whether the NPAR is related to the incidence rate of CKD.

CKD has gained significant attention due to aging. According to the Global Burden of Disease studies, CKD is now a leading cause of mortality worldwide [3]. Therefore, deciphering the underlying mechanisms of CKD and identifying targets for early diagnosis are crucial. The pathophysiology of CKD is complex and involves multiple reactions, with inflammation potentially playing a significant role [17]. Previous studies have shown that low-grade inflammation is a common characteristic of the uremic phenotype. Low-grade inflammation is associated with both CKD and its sequelae, such as protein-energy wasting and accelerated vascular aging, which can promote CVD and infections, ultimately leading to the death of CKD patients [17, 18]. Additionally, inflammation is involved in the renal injury and the development of renal fibrosis [19]. Targeting inflammatory factors can play a significant role in renoprotection [20].

The mechanistic relationship between NPAR and CKD remains to be elucidated. Inflammation is known to play a critical role in the development and progression of CKD [21]. In CKD, neutrophils are recruited by inflammatory mediators, which are secreted by injured tubular epithelial cells. Neutrophils then amplify the inflammation and promote further renal damage [22]. Besides, previous studies had proved that hypoalbuminemia was a risk factor for CKD progression to ESRD [23]. In fact, inflammatory biomarkers have been widely used in various clinical applications. A retrospective study identified neutrophil-to-lymphocyte ratio (NLR) as a potential predictor for distinguishing acute appendicitis and ureterolithiasis [24]. Previous studies have also reported that an increase in CRP levels was accompanied by a higher CKD morbidity rate and a rapid decline in eGFR [25]. Compared to these traditional inflammatory biomarkers, NPAR amplified the predictive value of inflammation and showed better clinical predictive value. Lan et al. found that NPAR performed a better role in predicting 5-year all-cause mortality of patients with chronic obstructive pulmonary disease [26]. This advantage was also demonstrated in predicting the occurrence of stroke-associated infection in patients with acute ischemic stroke [27]. Besides, Zhao et al. found that eGFR significantly decreases with increasing NPAR in pre-dialysis patients with stage 3–5 CKD and NPAR has a effectively predictive value for coronary atherosclerosis severity in CKD populations [28]. It was consistent with our research. These studies showed that NPAR has outstanding predictive ability in diseases. Furthermore, NPAR is a non-invasive easily accessible method, making it a promising option for clinical application [29].

This study has several strengths and limitations in this study. First, the sample we used was large and representative. Second, we adjusted for confounding factors to ensure that the associations were reliable. In addition, we used multivariate and subgroup analyses to further confirm the robustness of our findings. However, several limitations should be taken into consideration. First, due to the cross-sectional study design, we could not establish authentic causality. Second, NPAR was measured only once in our study, which may underestimate the association. Third, although we adjusted for several confounding factors, we could not completely exclude the influence of other potential covariates. Therefore, prospective studies with larger sample sizes are still needed to clarify the causal mechanism involved.

## 5 Conclusion

In conclusion, our study provides strong evidence supporting the association between elevated NPAR and CKD. This observation suggested that NPAR could be an available and cost-effective tool for early identifying and intervening the individuals at risk of CKD. Further research should aim to clarify the mechanisms that govern the relationship between NPAR and CKD.

## List of abbreviations

BMI: body mass index
BUN: blood urea nitrogen
CKD: chronic kidney disease
CVD: cardiovascular disease
DM: diabetes mellitus
eGFR: estimated glomerular filtration rate
HBP: high blood pressure
HDL: high-density lipoprotein
HGB: hemoglobin
LDL: low-density lipoprotein
NC: neutrophil count
NPAR: neutrophil-percentage-to-albumin ratio
PIR: poverty income ratio
TC: total cholesterol
TG: riglyceride
UA: uric acid
UACR: urinary albumin creatinine ratio
